# Positive Role of *Trichoderma harzianum* in Increasing Plant Tolerance to Abiotic Stresses: A Review

**DOI:** 10.3390/antiox14070807

**Published:** 2025-06-30

**Authors:** Yueyao Geng, Shuying Chen, Pinke Lv, Yankai Li, Jingxuan Li, Fangling Jiang, Zhen Wu, Qirong Shen, Rong Zhou

**Affiliations:** 1 Sanya Institute of Nanjing Agricultural University, Nanjing Agricultural University, Nanjing 210095, China; 2024804357@stu.njau.edu.cn (Y.G.); 14122318@stu.njau.edu.cn (S.C.); 14123204@stu.njau.edu.cn (P.L.); 2023204044@stu.njau.edu.cn (Y.L.); jxli@njau.edu.cn (J.L.); jfl@njau.edu.cn (F.J.); zpzx@njau.edu.cn (Z.W.); 2 Key Laboratory of Organic-Based Fertilizers of China, Key Laboratory for Solid Organic Waste Utilization, Nanjing Agricultural University, Nanjing 210095, China; shenqirong@njau.edu.cn; 3 Department of Food Science, Aarhus University, Agro Food Park 48, 8200 Aarhus, Denmark

**Keywords:** *T. harzianum*, abiotic stress, biological agents, regulatory mechanism

## Abstract

As a beneficial fungus, *Trichoderma harzianum* (*T. harzianum*) has been widely applied for growth promotion and biocontrol. Recently, it has attracted much attention with regard to improving stress tolerance in plants under abiotic stress. In this paper, the multiple mechanisms of *T. harzianum* for alleviating abiotic stress damage in plants are reviewed. *T. harzianum* can regulate the synthesis of key phytohormones, such as abscisic acid (ABA), indole-3-acetic acid (IAA), etc., thereby enhancing the physiological response ability of plants under stress conditions such as drought, salt stress, and high temperature. These are associated with antioxidant system regulation in plants, which reduces levels of reactive oxygen species (ROS) and oxidative damage and maintains intracellular redox balance. *T. harzianum* can also improve plant nutrient uptake and root development, secondary metabolism, soil environment and structure, and expression of related genes. In addition, in this paper, the characteristics of *T. harzianum* application in field and horticultural crops are summarized and compared, revealing differences in the methods, concentrations, time, and effects of applying *T. harzianum* to various crops. We further explore the synergistic regulation effect of *T. harzianum* and plant–microbiome interaction on the stress microenvironment. Future perspectives on the molecular mechanism of *T. harzianum* and its field application potential are discussed. This review provides a theoretic and practical reference for the application of *T. harzianum* in agricultural production.

## 1. Introduction

By 2030, nearly 600 million people are projected to be chronically undernourished and struggling to stay healthy [1]. Therefore, there is an urgent need to significantly increase agricultural productivity. However, with increases in extreme weather, abiotic stresses such as heat and drought can adversely affect the growth and development of plants, thereby reducing crop yield and quality [2].

The most important feature of drought stress is that ABA accumulation triggers a protective mechanism, leading to stomatal closure [3,4]. Stomatal closure reduces carbon dioxide absorption, which decreases photosynthesis and thereby inhibits plant growth and development [5]. Drought can also lead to the accumulation of peroxides, which can cause damage to cells [6]. Salt stress usually leads to disturbed ion absorption and hinders water transport, thus limiting plant growth and development [7]. High temperatures can cause plant pollen sterility, reduce stigma vitality, and significantly increase the levels of reactive oxygen species (ROS) in buckwheat flowers [8]. Furthermore, high temperatures lead to Rubisco inactivation and a decrease in the chloroplast electron transport rate, which reduces plant photosynthesis and inhibits growth [9]. These stressors not only interfere with the normal growth and metabolism of plants but also lead to a significant decline in crop yields, which seriously threatens global food security. Therefore, the development of effective strategies to improve the adaptability and productivity of crops under abiotic stress has become a top priority in agricultural science research and practice.

In recent years, biomicrobial agents have received a lot of attention as an innovative and sustainable solution to abiotic stresses. *T. harzianum* is a filamentous fungus belonging to the family *Carno-bacteriaceae* and *Xylaria*, which was first classified separately from *Trichoderma* by Rifai in 1969 and officially named *T. harzianum* [10]. This microorganism demonstrates the ability to colonize plant surfaces and form hyphal sheaths, effectively suppressing pathogenic bacteria while modulating the composition of fungal communities in the rhizosphere [11]. Its broad-spectrum resistance against various pests and diseases [12,13] has established it as a significant subject for research into plant biocontrol and growth promotion.

Recent research has consistently demonstrated the remarkable capacity of *T. harzianum* to enhance abiotic stress tolerance and improve agricultural productivity across a wide spectrum of economically important crops. Regarding vegetable crops, numerous controlled studies have validated its efficacy in tomato, where *T. harzianum* inoculation has been shown to significantly improve drought tolerance while simultaneously enhancing key growth parameters including plant height, leaf area, and root biomass [14,15,16]. Comparable beneficial effects have been well documented in other solanaceous crops such as cucumber [17] and eggplant [18], as well as perennial crops like black pepper [19]. Beyond horticultural species, this beneficial fungus has been proven equally effective in major cereal crops, with multiple independent studies confirming its growth-promoting effects in rice [20,21,22] and maize [23] under various stress conditions. More recent investigations have expanded the scope of *T. harzianum*’s applications to include important forage crops like Sudan grass [24] and oilseed crops such as Indian mustard [25], demonstrating its versatility across diverse agricultural systems. These consistent findings across multiple crops underscore the broad-spectrum potential of *T. harzianum* as a bio-enhancer in modern agriculture.

This review examines *T. harzianum* application methods in horticultural and field crops, along with its mechanisms for enhancing crop stress tolerance under abiotic stress conditions. The fungus can be applied through various methods including seed treatment, soil inoculation, foliar spraying, and root dipping, all of which have shown significant potential for improving plant growth and stress resilience. The mechanism of action is described in detail from six aspects: root development and nutrient absorption, secondary metabolism, antioxidant defense systems, plant hormone balance, soil structure, and expression of related genes. These multifaceted mechanisms collectively contribute to improved stress adaptation, significantly advancing our understanding of microbial-mediated plant stress tolerance and providing valuable insights for developing sustainable agricultural strategies under challenging environmental conditions. The integration of these findings with emerging multi-omics approaches promises to further elucidate the complex interactions between *T. harzianum* and crop plants, optimizing its application for enhanced agricultural productivity.

## 2. Methods of Applying *T. harzianum*

The effects of *T. harzianum* are wide-ranging; it can boosts resistance [26,27], control pathogens like *Fusarium acuminatum* [12] and *Fusarium wilt* [28], inhibit root-knot nematodes [13], improve aphid resistance (through RNA-mediated gene silencing) [29], and increase fruit antioxidant capacity and anthocyanin content [30]. As shown in Table 1, *T. harzianum* is applied to horticultural crops in a variety of ways that aim to enhance the symbiotic relationship between plants and microorganisms through early colonization. There are six main methods of applying *T. harzianum* to plants, including soil inoculation, seed treatment, root dipping, foliar spraying, addition to the irrigation system, and compost or organic fertilizer inoculation. The three most commonly used application methods for horticultural crops are seed treatment (tomato, eggplant) [15,16,18], soil application (cucumber, melon, black pepper) [17,19,27,28], and root treatments (strawberry, chrysanthemum) [30,31] (Table 1). Additionally, application times are diverse, in order to activate the plant defense mechanism in advance or to promote the development of the root system (Table 1).

Recently, research trends have tended to favor complex stress management and precise application strategies (e.g., regular irrigation for chrysanthemum [31]). In the future, the synergistic effect of *T. harzianum* and other probiotics can be further explored in combination with molecular means to analyze crop-specific response mechanisms, to optimize the effects of field application.

As shown in Table 2, the application of *T. harzianum* to field crops is mainly carried out through soil application (maize, Indian Mustard) [23,25], seed treatment (rice) [20,21,22], or root irrigation (Sorghum sudangrass, *Hordeum vulgare* L.) [24,32] with the optimal period being pre-sowing or at the transplanting period. These treatments significantly improve crop stress tolerance, modulate inter-root microbial communities, enhance antioxidant enzyme activities, and increase yield (Table 2).

Horticultural plants and field crops have both commonalities and differences with regard to the way *T. harzianum* is applied (Table 1 and Table 2). In terms of application methods, both types of crops may receive seed treatment, soil application, or root irrigation. However, horticultural plants are more inclined to receive seedling-stage treatment (Table 1). Application to field crops focuses more on seed treatment or soil application before sowing (Table 2). In terms of application effects, both types of crops showed significant effects relating to stress and growth promotion. Treatment of horticultural plants focuses more on quality enhancement and disease prevention and control. By comparison, those working with field crops have been more concerned with yield enhancement and stress tolerance enhancement. In addition, horticultural plants tend to receive multiple applications at critical growth periods, while field crops are mainly treated with a single treatment prior to planting or at transplanting (Table 1 and Table 2). These differences mainly stem from the differences in growth cycles, cultivation patterns, and types of stress between the two types of crops, and need to be optimized and adjusted to specific crop needs in practical applications.

The actual effect of *T. harzianum* can vary greatly depending on strain-specific characteristics, application methods, timing, concentration, and crop species. Since these factors together affect efficacy, follow-up studies need to combine these factors to select the most effective regimen. In addition, it is advisable to develop region-specific application guidelines to integrate local climate, soil conditions, and crop growth stages. This approach enables individualized biological control strategies to maximize the growth promotion and stress resistance potential of *T. harzianum*.

## 3. Mechanisms of *T. harzianum* Promotes Stress Tolerance of Plants Under Abiotic Stresses

### 3.1. Improving Plant Root Development and Nutrient Uptake

**Figure 1:**
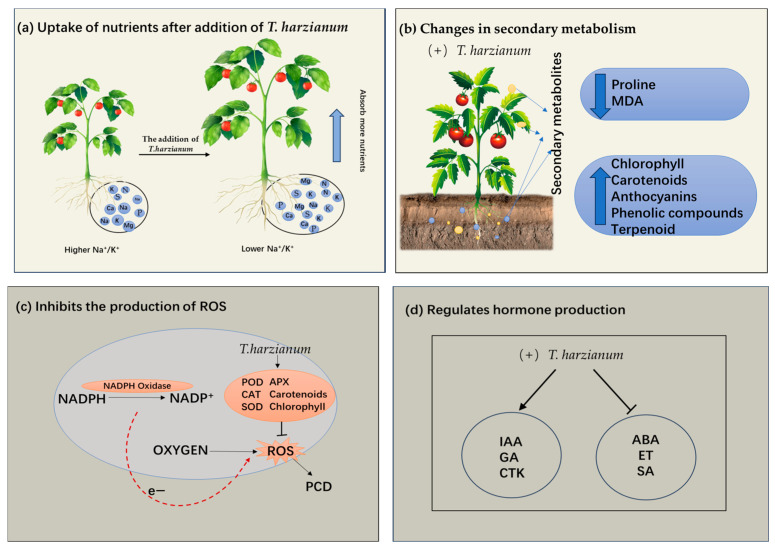
Four key mechanisms of *T. harzianum* to improve plant stress tolerance. (**a**) Promoting plant root growth and nutrient absorption. (**b**) Effect of *T. harzianum* on secondary metabolites in plants. (**c**) Enhancement of the plant’s antioxidant defense system. (**d**) Regulating the phytohormones. MDA: Malondialdehyde; ROS: Reactive oxygen species; PCD: Programmed cell death; POD: Peroxidase; APX: Ascorbate peroxidase; SOD: Superoxide dismutase; GA: Gibberellin; IAA: Indole acetic acid; ET: Endothelin; CTK: cytokinin; ABA: Abscisic acid; SA: Salicylic acid; CAT: Catalase.

The effects of *T. harzianum* on plant roots have been a key research focus [33]. Negative effects of abiotic stresses on roots are characterized by reductions in root length and weight, inhibition of root growth, and reductions in root surface area and nutrient uptake. *T. harzianum* ameliorates injuries from abiotic stresses (Figure 1a). Under salt stress, the total root length of barley cultivars Gairdner and Vlamingh cultivars inoculated with *T. harzianum* increased [32]. Under drought stress, *T. harzianum* promoted lateral root branching and increased total root length from 32.5% to 42.7% [26]. In addition, peptaibol secreted by *T. harzianum* increased the fresh weight of pepper roots by enhancing membrane stability and osmotic regulation [19]. *T. harzianum* treatment increased proline accumulation by 50%, upregulated *CsAPX* expression, and improved salt tolerance in cucumber via promoting lateral root development [17].

Studies have shown that organic acids (such as oxalic acid, citric acid) secreted by *T. harzianum* can reduce soil pH and dissolve insoluble phosphorus and potassium, thereby releasing available nutrients such as phosphorus and potassium [34]. The concentrations of nutrients (N, P, S, Ca, Mg, and K) were significantly increased under different *Trichoderma* treatments, and the Na/K ratio was significantly reduced, mainly due to increased K absorption [25]. In addition, *T. harzianum*, as a species of *Trichoderma*, can produce various substances to promote plant growth, such as ethylene (Ethylene), and IAA to stimulate root development, and the hydrophobins and swollenin produced by *Trichoderma* assist in its colonization of plant roots [35]. When plants encounter salt stress via their roots, *T. harzianum* initiates a sophisticated protective response by modulating ion homeostasis, which facilitates the selective accumulation of beneficial ions and promotes the synthesis of compatible osmolytes, thereby effectively reducing ionic toxicity and maintaining cellular turgor pressure [17].

*T. harzianum* has shown great potential for use in promoting plant root absorption and development, enhancing stress resistance, and improving soil microecology. It has enormous potential for the future development of sustainable agriculture, ecological restoration, and smart agriculture.

### 3.2. Enhancement of Plant Secondary Metabolism

Recent studies have confirmed that levels of plant secondary metabolite accumulation significantly correlate with abiotic stress tolerance [36,37]. *T. harzianum* can enhance plant tolerance by modulating the levels of plant secondary metabolites (Figure 1b). In black pepper, *T. harzianum* inoculation under drought stress reduced proline accumulation and improved biomass yield compared with *T. asperellum* [19]. This interaction reduces lipid peroxidation, as evidenced by 40% lower malondialdehyde (MDA) levels in *Trichoderma*-colonized cucumber under salt stress compared with untreated plants [17]. *T. harzianum* also enhances stress resistance through broader metabolic reprogramming, including increased other synthesis and secondary metabolites such as phenol and flavonoid complexes [26]. *T. harzianum* also induces changes in terpenoid content in plants [38]. By modulating these metabolites, *T. harzianum* enhances plant resilience to multiple abiotic stressors.

The contents of chlorophyll a, chlorophyll b, carotenoids, and anthocyanins increased after inoculation with *T. harzianum*, and the maintained photosynthetic efficiency to varying degrees compared to the salt-stressed plants that were not inoculated with *T. harzianum* under 150 mM NaCl treatment [39]. This suggests that *T. harzianum* helps maintain photosynthesis in plants and supports normal growth and development under salt stress. Collectively, these findings demonstrate that *T. harzianum* enhances plant abiotic stress tolerance through multifaceted metabolic and physiological adjustments. This metabolic reprogramming not only improves plant survival under adverse conditions but also supports sustained growth and productivity, highlighting its potential as a powerful bioinoculant for stress-resilient agriculture.

### 3.3. Enhancement of Plant Antioxidant Defense System

Under abiotic stress, ROS in plants are altered [40]. ROS are reactive oxygen molecules in cells, and an excess of ROS leads to oxidative stress, which in turn triggers programmed cell death (PCD). NADPH oxidases play a pivotal role in plant growth and development by catalyzing the production of reactive oxygen species (ROS). These ROS act as signaling molecules that regulate various cellular processes essential for growth, including cell expansion. For instance, in Arabidopsis roots, the NADPH oxidase RHD2 generates ROS that are required to sustain root hair elongation [41]. Genetic mutation of RHD2 or pharmacological inhibition of NADPH oxidase activity with diphenylene iodonium (DPI) reduces ROS levels, resulting in a short-root-hair phenotype, demonstrating the essential role of NADPH oxidase-derived ROS in root hair growth regulation [41].

*T. harzianum* induces increased expression of antioxidant enzyme genes and elevated activity of antioxidant enzymes such as superoxide dismutase (SOD), peroxidase (POD), catalase (CAT), etc., in plant cells, which reduces the level of ROS [17] (Figure 1c). Similar increases in POD and APX activity were observed in *Trichoderma*-treated Indian mustard under saline conditions and were associated with improved yield and stress tolerance [25]. As further evidence, inoculation of *T. harzianum* reduced H_2_O_2_ levels in cucurbits by 10% under salt stress, effectively reducing oxidative damage [39]. These results consistently suggest that *T. harzianum* plays a central protective role in plants in response to abiotic stresses by regulating the enzyme antioxidant defense system.

Moreover, *T. harzianum* increased chlorophyll and carotenoid levels by 30% in *Satureja hortensis* under salt stress, thereby maintaining photosynthetic efficiency and ROS homeostasis [38]. Preservation of chlorophyll ensures continued light harvesting under stress, while carotenoids act as antioxidants and reduce lipid peroxidation [26]. Thereby, *T. harzianum* can improve plant stress tolerance via both enzyme and non-enzyme antioxidant defense systems.

### 3.4. Regulation of Plant Hormone Balance

Phytohormones are important for various aspects of plant growth and development, such as cell division, elongation, and differentiation, as well as plant budding, rooting, and flowering [42]. Plants are subjected to a variety of abiotic stresses that elicit different responses to defend against external abiotic stresses, and phytohormones are the most important endogenous subunits that regulate physiological and molecular responses [43] which can enhance resistance under abiotic stresses. For example, auxin can help plants maintain growth and metabolic homeostasis by regulating the expression of recombinant genes under drought and salt stress [44].

Studies have shown that plant hormones regulate abiotic stress response through complex interaction networks and molecular mechanisms, while microorganisms (such as *Trichoderma*) can enhance plant stress resistance through multitarget intervention (Figure 1d). This versatile fungus enhances plant performance through multiple physiological pathways. It stimulates the production of IAA, a crucial phytohormone [22,26,27]. As an important phytohormone, ethylene is widely involved in regulating plant growth, development, and senescence [45]. Lower concentrations of ethylene in the root zone significantly promote bud growth and biomass accumulation [46]. *T. harzianum* reduces ethylene biosynthesis by secreting ACC deaminase, thereby alleviating the inhibitory effect of ethylene on plant growth [47]. Research also revealed that *T. harzianum* can promote the production of salicylic acid (SA) and cytokinin (CTK). When tomato is subjected to abiotic stress, *T. harzianum* enhances the synthesis of growth hormones such as IAA, gibberellin (GA), and indolebutyric acid (IBA) [26]. *T. harzianum* protects cell membranes from ROS damage by regulating the secondary metabolism and promotes root nutrient uptake, thereby synergistically enhancing plant stress resistance and growth [26]. Similarly, Contreras-Cornejo et al. found that *T. harzianum* could enhance the antioxidant defense system of plants by regulating endogenous hormones and gene expression to alleviate adversity stress, by investigating the mechanism of *T. harzianum* action under different abiotic stresses [48]. When the *Thkel1* (transketolase-like 1) in *T. harzianum* was overexpressed in *Arabidopsis thaliana*, ABA levels in transgenic Arabidopsis were significantly lower than those in wild plants, which was more pronounced under salt stress [49]. Expression of the *Thkel1* in *Arabidopsis thaliana* enhances plant tolerance to salt and osmotic stress, accompanied by increased glucosidase activity and decreased abscisic acid (ABA) levels compared to those observed in wild-type plants [49].

### 3.5. Improvement of Soil Environment and Structure

**Figure 2:**
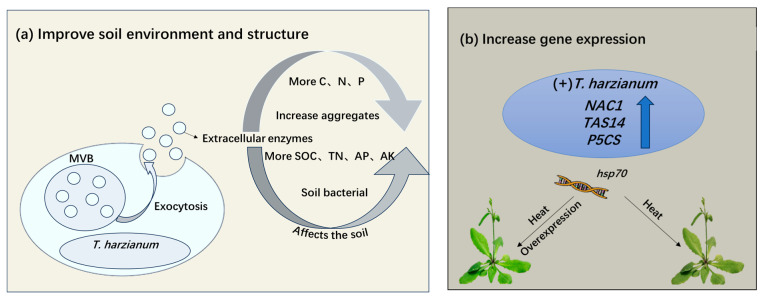
*T. harzianum* enhances plant adaptability to abiotic stress by improving soil environment and regulating stress resistance genes. (**a**) Improvement of soil environment and crop structure. (**b**) Altering the expression of related genes that enhance plant resistance under abiotic stresses by *T. harzianum*. MVB: Multivesicular body; SOC: Soil organic carbon; TN: Total nitrogen; AP: Available phosphorus; AK: Available potassium; hsp70 (heat-shock protein 70), TAS14 inducible by ABA (abscisic acid) and environmental stress; NAC1 (NAM, ATAF1/2, and CUC2); P5CS (delta 1-Pyrroline-5-Carboxylate Synthetase).

Abiotic stress is a stress condition that not only causes direct harm to plants but also alters the biochemical and structural properties of soils [50]. *T. harzianum* can alleviate the negative effects of abiotic stress on soil through multiple mechanisms to improve plant stress tolerance (Figure 2a). *T. harzianum* can secrete a variety of extracellular enzymes, such as protease, cellulase, ligninase, hemicellulase, etc., and gradually decompose complex or organic macromolecules into simple organic small molecules [51]. This releases carbon, nitrogen, phosphorus, and other elements in the soil for plants to absorb and utilize, and enhances soil fertility. *T. harzianum* can also significantly increase the content of large aggregates (>2 mm) and small aggregates (0.25–2 mm) in soil and improve the stability of soil aggregates. In one reported study, the contents of soil organic carbon, total nitrogen, available phosphorus, and available potassium increased [52]. The soil bacterial community structure was significantly changed by *T. harzianum* application, with increased relative abundance of *Proteobacteria* and *Actinobacteria* and decreased relative abundance of *Acidobacteria* and *Chloroflexi*, providing evidence for plant-*Trichoderma* interactions [52,53].

*T. harzianum* effectively alleviates the adverse effects of abiotic stress on the soil–plant system by secreting various extracellular enzymes to decompose soil organic matter, improve aggregate structure, and regulate microbial communities. Future research should focus on elucidating the mechanisms by which its extracellular polysaccharides mediate soil structure restoration, developing synergistic microbial agents with functional microorganisms, optimizing application techniques for different soil–crop systems, and assessing its long-term ecological effects under extreme climatic conditions. These efforts aim to advance the large-scale application of this green biotechnology in soil remediation and sustainable agriculture.

### 3.6. Expression Analysis of the Hsp70 (Heat-Shock Protein 70), TAS14 Inducible by ABA and Environmental Stress, NAC1 (NAM, ATAF1/2, and CUC2) and P5CS (Delta 1-Pyrroline-5-Carboxylate Synthetase) Genes

Overexpression of the *hsp70* gene in *T. harzianum* has emerged as a promising strategy to increase plant tolerance to various abiotic stresses [54,55]. In addition, *hsp70* overexpression confers cross-tolerance to drought and salinity in plants by stabilizing cellular proteins and regulating ion homeostasis through interactions with stress-responsive pathways, such as SOS1-mediated Na^+^/H^+^ exchange [56].

The role of *Trichoderma*-derived genes in plant stress acclimation is not limited to *hsp70*. For example, inoculation of tomato plants with *T. harzianum* induced the expression of the *NAC1*, *TAS14*, and *P5CS* genes under cold stress conditions, with a 2.1-to-2.3-fold increase in the transcriptional level of *P5CS* [15] (Figure 2b). As important transcriptional regulators, NAC family genes are involved in plant development regulation and mediate adaptive responses to abiotic stresses [57]. *Arabidopsis thaliana* lines overexpressing *NAC1* gene showed significantly improved plant survival under cold stress [58]. Overexpression of *TAS14* enhanced plant tolerance to abiotic stresses such as drought and salt stress [59]. *P5CS* is a key gene for proline biosynthesis, which is accumulated in excess to enhance plant stress tolerance [60]. Research has demonstrated that *T. harzianum* treatment enhances drought tolerance of rice by modulating the expression of key genes involved in photosynthesis, antioxidant activity, osmotic regulation, and phytohormone signaling under drought stress [21]. These findings indicate that *T. harzianum* improves plants’ resilience to multiple abiotic stresses through transcriptional regulation.

In the future, it will be possible to validate the function of stress-resistant genes in major crops through cross-species verification and develop molecular marker-assisted breeding technologies. Synthetic biology methods can be used to construct multi-gene co-expression systems, which can be combined with gene editing technologies to optimize plant stress resistance. Finally, the agronomic stability of gene regulation can be assessed through multi-environment stress field trials, providing new targets and multi-dimensional breeding strategies for crop stress resistance and genetic improvement.

## 4. Conclusions

In conclusion, this review provides a robust theoretical foundation for *T. harzianum* application and insights into the molecular mechanisms of *T. harzianum*-mediated plant abiotic stress responses. *T. harzianum*, as an important promoting plant growth fungus, has shown remarkable effects in enhancing plant resistance to abiotic stresses such as drought, salinity, and extreme temperature. Its mechanism of action mainly includes improving the root environment, activating the plant antioxidant defense system, reducing the accumulation of reactive oxygen species, coordinating the plant hormone signaling network, etc., and optimizing the stress response. These synergistic effects significantly mitigate the adverse effects of stress on plant growth and development. Its multifaceted mechanisms make *T. harzianum* an exceptionally valuable biological tool for enhancing crop performance under challenging environmental conditions.

Although *T. harzianum* has made important progress in the application of abiotic stress, there are still some key scientific problems in this field that need to be solved urgently. First, there are significant differences in the stress response mechanisms of different genotypes of *T. harzianum*, and the molecular basis of its interaction with plants remain unclear. Secondly, the functional stability and regulatory network of strains under compound stress still need to be systematically analyzed. In view of these problems, future research should focus on the integration of multiomics technologies (genomics, transcriptomics, proteomics, etc.) to reveal strain-specific stress response mechanisms. In addition, it is necessary to develop efficient gene editing tools to improve key functional genes (such as antioxidant enzyme genes and osmoregulation-related genes). It is also necessary to optimize the formulation and application technology of the fungus agent to improve the colonization efficiency and application effect in the complex environment of the field. The study provides a robust theoretical foundation for *T. harzianum* application and insights into the molecular mechanisms of *T. harzianaum*-mediated plant stress responses.

## Figures and Tables

**Table 1:** Method, amount, time and effect of application of *T. harzianum* to horticultural crops.

Plant Species	Method	Amount	Time	Effect	Reference
Tomato	Seed immersion as well as seedling treatment or sprinkled under the surface of the soil	10^7^ CFU/mL or 10 g/pot	Before sowing and before stress treatment or during the growing period	Mitigates low-temperature and water-deficit stress, and promotes growth	Ghorbanpour et al., 2017 [15], Khoshmanzar et al., 2020 [16]
Cucumber	Root drenching or soil application	10^8^ spores mL^−1^ or 10^4^–10^7^ CFU/g	Before salt stress or before sowing	Enhances plant physiology and biochemistry, controls wilt and improves yield and quality	Zhang et al., 2019 [17], Lian et al., 2023 [27]
Eggplant	Seed treatment (seed germination)	4.9 × 10^8^ CFU/L	Before the seed germination experiment	Significantly enhanced seed germination, seedling growth, and photosynthesis	Wu et al., 2017 [18]
Melon	Add to mixtures containing peat and vermiculite	10^6^ conidia g^−1^ of peat	Seedling stage and before transplanting	Increase plant fresh weight, promote growth, and inhibit wilt	Martínez-Medina et al., 2011 [28]
Strawberry	Pre-transplant root dipping and post-transplant root irrigation	10^7^ spores/mL	Before and after transplanting	Regulates physiological processes, improves fruit yield and quality	Lombardi et al., 2020 [30]
Black Pepper	Soil application	10 g/bag for soil	After drought treatment	Enhance physiological and biochemical responses and alleviate water stress	Valiyambath et al., 2024 [19]
Chrysanthemum	Root irrigation	10^6^ CFU/mL	At the time of cutting, then once every five days	Improve the rooting quality of chrysanthemum cuttings	Wu et al., 2024 [31]

**Table 2:** Method, amount, time, and effect of application of *T. harzianum* to field crops.

Plant Species	Method	Amount	Time	Effect	Reference
Maize	Soil application	10^7^ CFU/mL soil	Before sowing	Alterations in maize growth, inter-root microbial communities and biological control of *Fusarium* stem rot	Saravanakumar et al., 2017 [23]
Rice	Seed treatment (seed coating) or root dipping	10 g/kg seeds or 1–3 × 10^5^ CFU/mL	Before sowing or at transplanting	Improves drought tolerance, enhances antioxidant enzyme activity, increases water use efficiency and yield	Bashyal et al., 2020 [20] Bashyal et al., 2021 [21] Pandey et al., 2016 [22]
Sorghum sudangrass	Root irrigation	10^6^ CFU/mL	Before the treatment of pathogens in the seedling stage	Prevention and control of leaf spot disease caused by anthrax	Han et al., 2024 [24]
Indian Mustard	Soil application (mixed with fertilizer) or soak with suspended seeds	7.5–12.5 g/kg soil or 1.1 × 10^5^–10^7^ CFU	Before sowing	Improves salt tolerance, enhances antioxidant enzyme activity, increases seed yield under salt stress	Saha et al., 2024 [25]
*Hordeum vulgare* L.	Add spore suspension to the roots of the seedlings	10^8^ spores/mL	Before salt treatment	Alters the metabolome and lipidome of barley to enhance its tolerance to salt stress	Gupta et al., 2021 [32]

## Data Availability

Not applicable.
